# The dopamine D_1_ receptor is expressed and facilitates relaxation in airway smooth muscle

**DOI:** 10.1186/1465-9921-14-89

**Published:** 2013-09-02

**Authors:** Kentaro Mizuta, Yi Zhang, Dingbang Xu, Fumiko Mizuta, Frank D’Ovidio, Eiji Masaki, Charles W Emala

**Affiliations:** 1 Departments of Anesthesiology, College of Physicians and Surgeons of Columbia University, 630W 168th St, P&S Box 46, New York, NY 10032, USA; 2 Departments of Surgery, College of Physicians and Surgeons of Columbia University, 622W 168th St, PH 14, Room 104, New York, NY 10032, USA; 3 Department of Dento-oral Anesthesiology, Tohoku University Graduate School of Dentistry, 4-1 Seiryo-machi, Aoba, Sendai, Miyagi 9808575, Japan

**Keywords:** Dopamine, RT-PCR, Immunoblot, G_s_-coupled receptor, Cyclic AMP, PKA, Epac

## Abstract

**Background:**

Dopamine signaling is mediated by G_s_ protein-coupled “D_1_-like” receptors (D_1_ and D_5_) and G_i_-coupled “D_2_-like” receptors (D_2-4_). In asthmatic patients, inhaled dopamine induces bronchodilation. Although the G_i_-coupled dopamine D_2_ receptor is expressed and sensitizes adenylyl cyclase activity in airway smooth muscle (ASM) cells, the G_s_-coupled dopamine D_1_-like receptor subtypes have never been identified on these cells. Activation of G_s_-coupled receptors stimulates cyclic AMP (cAMP) production through the stimulation of adenylyl cyclase, which promotes ASM relaxation. We questioned whether the dopamine D_1_-like receptor is expressed on ASM, and modulates its function through G_s_-coupling.

**Methods:**

The mRNA and protein expression of dopamine D_1_-like receptor subtypes in both native human and guinea pig ASM tissue and cultured human ASM (HASM) cells was measured. To characterize the stimulation of cAMP through the dopamine D_1_ receptor, HASM cells were treated with dopamine or the dopamine D_1_-like receptor agonists (A68930 or SKF38393) before cAMP measurements. To evaluate whether the activation of dopamine D_1_ receptor induces ASM relaxation, guinea pig tracheal rings suspended under isometric tension in organ baths were treated with cumulatively increasing concentrations of dopamine or A68930, following an acetylcholine-induced contraction with or without the cAMP-dependent protein kinase (PKA) inhibitor Rp-cAMPS, the large-conductance calcium-activated potassium (BK_Ca_) channel blocker iberiotoxin, or the exchange proteins directly activated by cAMP (Epac) antagonist NSC45576.

**Results:**

Messenger RNA encoding the dopamine D_1_ and D_5_ receptors were detected in native human ASM tissue and cultured HASM cells. Immunoblots confirmed the protein expression of the dopamine D_1_ receptor in both native human and guinea pig ASM tissue and cultured HASM cells. The dopamine D_1_ receptor was also immunohistochemically localized to both human and guinea pig ASM. The dopamine D_1_-like receptor agonists stimulated cAMP production in HASM cells, which was reversed by the selective dopamine D_1_-like receptor antagonists SCH23390 or SCH39166. A68930 relaxed acetylcholine-contracted guinea pig tracheal rings, which was attenuated by Rp-cAMPS but not by iberiotoxin or NSC45576.

**Conclusions:**

These results demonstrate that the dopamine D_1_ receptors are expressed on ASM and regulate smooth muscle force via cAMP activation of PKA, and offer a novel target for therapeutic relaxation of ASM.

## Background

Dopamine is a ubiquitous endogenous catecholamine neurotransmitter in the mammalian central nervous system, where it controls a variety of physiological responses including emotion, cognition, and endocrine functions [1-3]. This catecholamine also modulates cardiovascular function, hormone secretion, vascular tone, renal function, and gastrointestinal motility through the specific dopamine receptor subtypes expressed in peripheral organs and tissues [3-7]. The dopamine receptor is a member of the large family of 7 transmembrane spanning domain G protein-coupled receptors (GPCRs). The five known subtypes of dopamine receptors have been subdivided into two distinct subclasses, “D_1_-like” receptors (D_1_ and D_5_) which couple to the G_s_ protein and “D_2_-like” receptors (D_2_, D_3_ and D_4_) which couple to G_i_[3,8].

Inhaled dopamine induces bronchodilation during bronchial obstruction in asthmatic patients [9-11]. The dopamine-induced bronchodilation has been thought to be mediated via β_2_-adrenoceptors because this effect is partially blocked by the β-adrenoceptor antagonist propranolol [12]. However, a recent study suggested that the dopamine D_1_-like receptor antagonist SCH23390 inhibited the bronchodilator effect of dopamine in acetylcholine-contracted rat tracheal rings suspended in organ baths [13], suggesting that the dopamine D_1_-like receptor is expressed on airway smooth muscle and induces bronchodilation. Although chronic activation of the dopamine D_2_ receptor has been shown to sensitize adenylyl cyclase and facilitate forskolin-induced airway smooth muscle relaxation [14], the functional expression of the dopamine D_1_-like receptor subtype on airway smooth muscle has not been described.

Activation of G_s_-coupled receptors leads to stimulation of adenylyl cyclase activity resulting in an increase in cellular cyclic AMP (cAMP) levels. Increase in intracellular cAMP levels activates cAMP-dependent protein kinase (PKA). PKA has many intracellular targets including the phosphorylation of myosin light chain kinase (MLCK), which classically promotes airway smooth muscle relaxation [15,16]. In addition, exchange proteins directly activated by cAMP (Epac) have recently been identified on airway smooth muscle as novel cAMP sensors able to elicit airway smooth muscle relaxation in human and guinea pig [17,18]. These findings led us to hypothesize that functional dopamine D_1_-like receptors could respond to dopamine in airway smooth muscle and promote its relaxation through cellular cAMP’s activation of multiple potential signaling pathways.

In the present study, the expression of the dopamine D_1_-like receptor was assessed in native human and guinea pig airway smooth muscle tissue and cultured human airway smooth muscle (HASM) cells. In addition, cAMP production and dopamine D_1_ receptor effects on ex vivo contractile tone were assessed to confirm its physiological role in airway smooth muscle.

## Materials and methods

### Materials

M-199 smooth muscle medium, human fibroblast growth factor, human epidermal growth factor, fetal bovine serum (FBS), and antibiotic-antimycotic were purchased from Life Technologies (Grand Island, NY). Lysates of human brain cerebral cortex and kidney used as positive protein controls on immunoblots were obtained from BD Biosciences (Palo Alto, CA). Protease inhibitor cocktail III was purchased from EMD Biosciences (San Diego, CA). A68930 and SCH39166 were purchased from Tocris Bioscience (Ellisville, MO). NSC45576 was a kind gift from the Open Clinical Repository of the National Cancer Institute’s Developmental Therapeutics Program. HitHunter™ cAMP XS+ assay kit was purchased from DiscoveRx (Fremont, CA). All other chemicals were obtained from Sigma (St. Louis, MO) unless otherwise stated.

### Cell culture

Cultures of HASM cells came from three sources. Primary cultured HASM cells were obtained from (1) Lonza (Walkersville, MD), and (2) were a kind gift from Dr. Reynold A Panettieri, Jr. (University of Pennsylvania, PA), which were obtained from lung transplant donors in accordance with procedures approved by the University of Pennsylvania Committee on Studies Involving Human Beings as previously described [19]. (3) Cultures of immortalized HASM cells were a kind gift from Dr. William Gerthoffer (University of South Alabama, Mobile, AL) and have previously been characterized [20]. Cells were grown in culture medium M-199, supplemented with 10% FBS, 1 ng/ml human fibroblast growth factor, 250 pg/ml human epidermal growth factor, and an antibiotic-antimycotic mix (100 units/ml penicillin G sodium, 100 μg/ml streptomycin sulfate, 0.25 μg/ml amphotericin B) at 37°C in 95% air/5% CO_2_.

### Isolation of smooth muscle from human and guinea pig trachea

All Studies were approved by Columbia University’s Institutional Review Board (IRB) and deemed not human subjects research under 45 CFR 46. Human trachea was obtained from discarded regions of healthy donor lungs harvested for lung transplantation at Columbia University. Human tissue was transported in cold buffer as described [14].

All guinea pig studies were approved by the Columbia University Institutional Animal Care and Use Committee. These studies were also reviewed by the Committee on the Ethics of Animal Experiments in Tohoku University School of Medicine, and they were carried out in accordance with both the Guidelines for Animal Experiments issued by the Tohoku University and The Law (No. 105) and Notification (No. 6) issued by the Japanese Government. Adult male Hartley guinea pigs (approx. 400 g body weight) were deeply anesthetized by intraperitoneal pentobarbital (50 mg/kg). The chest cavity was opened, and the guinea pigs were exsanguinated. The entire trachea and whole brain (positive control for immunoblot) were surgically removed and immersed in cold (4°C) KH buffer. The exteriors of either the human or guinea pig trachea were carefully dissected free of adherent connective tissue under a microscope. Epithelium was left intact for immunohistochemistry but removed for organ bath experiments and immunoblotting.

### Isolation of RNA and reverse transcriptase polymerase chain reaction

Total RNA was extracted from freshly dissected native human airway smooth muscle, and primary cultured HASM cells (Lonza, Walkersville, MD) using TRIzol Reagent (Ambion, Austin, TX) according to the manufacturer’s recommendations. Total RNA from whole human brain (Clontech, Mountain View, CA) was used as a positive control. The extracted RNA from freshly dissected native human airway smooth muscle and primary cultured HASM cells were treated enzymatically with DNase I (Life Technologies, Grand Island, NY) to digest contaminating genomic DNA according to the manufacturer’s instructions. cDNA was synthesized using SuperScript VILO cDNA Synthesis Kit (Life Technologies, Grand Island, NY). Briefly, 1 μg of DNase I-treated total RNA was reverse transcribed at 42°C. The reaction was terminated by heating to 85°C for 5 min and then cooled at 4°C.

PCR was performed and analyzed as described (1) with sense and antisense primers corresponding to the “D_1_ subgroup (D_1_ and D_5_) of dopamine receptors (Table 1). Two-step PCR (annealing and extension at same temperature) was performed with Mastercycler ep gradient S thermal cycler (Eppendorf, Hamburg, Germany) for all PCR reactions and all reactions included an initial denaturation step at 94°C for 1 min followed by 40 cycles of denaturation (94°C for 10 s) and annealing/extension at 72°C for 1 min. Dopamine D_1_ primers were designed to anneal in two separate exons spanning a large intron to ensure that amplified PCR products resulted from amplified cDNA and not contaminating genomic DNA. Since the dopamine D_5_ receptor gene is intronless, complete removal of genomic DNA from the RNA samples was verified by performing negative control reactions in which reverse transcriptase was omitted from the cDNA synthesis reaction (-RT controls). PCR products were electrophoresed on 5% nondenaturing polyacrylamide gel in Tris, acetate, EDTA buffer. The gel was stained with ethidium bromide, and visualized using ultraviolet illumination. The gel image was captured with a model Powershot A570 digital camera (Canon, Tokyo, Japan).

**Table 1:** **primer sequences for dopamine D**_**1**_**-like receptor subtypes**

**Target**	**Sequence of primer**	**Amplicon size (bp)**
**(GenBank access no.)**		
Human Dopamine D_1_	FP: 5′-CCA GCG AAG TCC ACA TTC CAA GCT CC-3′	207
(NM_000794)	RP: 5′-GCC TCT GCT CTG CTA GTC AGT TGC AAT CAC-3′	
Human Dopamine D_5_	FP: 5′- CTG GTG TGC GCA GCC ATC GT-3′	173
(NM_000798)	RP: 5′- ACC CAG ACG TCG CAG AAC GC-3′	

*FP* forward primer, *RP* reverse primer.

### Immunoblot analysis

Confluent cultures of HASM cells were rinsed with cold phosphate-buffered saline (PBS), and mechanically scraped from the surface of the T75 culture flask in the presence of protease inhibitor cocktail III. Cells were pelleted (500 *g*, 10 min, 4°C) and lysed in cold lysis buffer [50 mM Tris–HCl, pH 8.0, 1% Nonidet P-40, 0.5% sodium deoxycholate, 0.1% SDS, 150 mM NaCl, 1 mM EDTA, 1:200 dilution of protease inhibitor cocktail III, 1 mM Na_3_VO_4_, 1 mM NaF]. Lysed cells were centrifuged (15,000 *g*, 15 min, 4°C) and an aliquot of the supernatant was subjected to protein analysis and storing at -80°C. Freshly dissected native human and guinea pig airway smooth muscle, and guinea pig brain cerebral cortex was homogenized (Tekmar Ultra Turrax T25 high-speed homogenizer set at top speed for 30 s) in cold (4°C) buffer (50 mM Tris, 10 mM HEPES, pH 7.4, 1 mM EDTA with a 1:200 dilution of protease inhibitor cocktail III). The homogenate was filtered through 125-μm Nitex mesh and centrifuged twice at 500 *g* for 15 min. The supernatant was transferred into new tubes and centrifuged at 50,000 *g* for 30 min at 4°C. The final membrane pellet was resuspended in the same buffer for protein concentration determinations and stored at -80°C. Each sample was solubilized by heating at 95°C for 10 min in sample buffer before use.

All the samples and positive controls were electrophoresed (8% SDS-PAGE) and transferred to PVDF membranes. 5% membrane blocking agent (GE Healthcare, RPN2125V, Waukesha, WI) in Tris-buffered saline with 0.1% Tween 20 (TBST) was used to block non-specific binding for 1 hr at room temperature. Membranes were then probed with antibodies directed against the dopamine D_1_ receptor protein (rabbit monoclonal 1:1,000; Epitomics, 2192-1, Burlingame, CA) or the dopamine D_5_ receptor protein (rabbit polyclonal 1:500; Santa Cruz, sc-25650, Santa Cruz, CA) overnight at 4°C. After washing three times with TBST, membranes were incubated for 1 hr at room temperature with HRP-labeled secondary anti-rabbit antibodies (1:5,000; GE Healthcare, NA934V, Waukesha, WI). The signals from the immunoreactive bands were detected by ECL Plus (GE Healthcare, Waukesha, WI) and were captured using chemiluminescent image analyzer (LAS 4000 Mini; GE Healthcare, Waukesha, WI).

### Immunohistochemistry

Human and guinea pig tracheal rings were fixed, paraffin embedded, sectioned and rehydrated as previously described [14]. Heat-mediated antigen retrieval, blockade of endogenous peroxidase, protein blocking and avidin-biotin blocking was performed as described [14] and slides were incubated overnight at 4°C in primary antibody against the dopamine D_1_ receptor protein (rabbit monoclonal 1:2,000; Epitomics, 2192-1, Burlingame, CA) or dopamine D_5_ receptor protein (rabbit polyclonal 1:2,000; Santa Cruz, sc-25650, Santa Cruz, CA), in 2% normal goat serum in PBS with 0.1% Triton X-100 (PBST). A parallel tracheal ring section was incubated with an isotype-specific rabbit IgG antibody as a negative control. After overnight incubation at 4°C, slides were washed three times with PBST and primary antibodies were detected using biotinylated anti-rabbit antibodies (Vector Laboratories, Peterborough, UK) at a concentration of 1:100. After incubation with ABC-HRP complex (Vector Laboratories, Peterborough, UK) for 30 min, the antigen antibody complex was then visualized with the peroxidase substrate kit (DAB) (SK-4100, Vector Laboratories, Peterborough, UK). Sections were counterstained with hematoxylin (Vector Laboratories, Peterborough, UK), dried, dehydrated in a graded alcohol series and xylene, and cover slipped using Poly-mount (Polysciences, Warrington, PA).

### cAMP assays

The dopamine D_1_ receptor-mediated cAMP production in primary cultured HASM cells (Lonza; Walkersville, MD) was measured using a HitHunter™ cAMP XS+ Assay kit according to the manufacturer’s instructions. Briefly, HASM cells in white 96-well plates were washed twice with warm PBS (37°C). The cells were incubated with dopamine (1 μM), or the dopamine D_1_-like receptor agonists (A68930 or SKF38393) (1 μM) for 20 min at 37°C. To examine the effect of dopamine on cAMP production in the presence of dopamine D_2_ receptor blockade, the cells were pretreated with the dopamine D_2_ receptor antagonist L-741626 (1 μM) for 30 min followed by incubation with dopamine (1 μM) for 20 min at 37°C. Incubation of the cells with L-741626 (1 μM) alone was also performed to determine whether L-741626 by itself exerts any effect on cAMP production. To examine the dose-dependent and time-course effects of the dopamine D_1_-like receptor agonist A68930 on cAMP production, the cells were incubated with A68930 (1 nM - 100 μM) for 10 - 60 min at 37°C. In separate experiments, the cells were pretreated with the dopamine D_1_-like receptor antagonists [SCH23390 (1 μM) or SCH39166 (1 μM)] or vehicle (PBS) for 30 min followed by incubation with A68930 (1 μM) for 20 min at 37°C. Then cAMP XS antibody reagent followed by the mixture of enzyme donor (ED)/Lysis/chemiluminescence (CL) working solution was added to each well. After incubation for 60 min at room temperature, cells were further incubated with the enzyme acceptor (EA) reagent for 3 hrs at room temperature. Luminescence signals were detected using a multimode microplate reader (Appliskan, Thermo Fisher Scientific, Waltham, MA).

### In vitro assessment of dopamine D_1_ receptor agonist effect on guinea pig airway smooth muscle relaxation after contraction with acetylcholine

Guinea pig tracheas were dissected and equilibrated in organ baths as described [21]. All rings were precontracted with 10 μM N-vanillylnonanamide (capsaicin analogue) and then 2 cycles of cumulatively increasing concentrations of acetylcholine (0.1 μM - 1 mM) with extensive buffer washes and resetting of the resting tension to 1.0 g between cycles. Rings were pretreated with 1 μM tetrodotoxin and 10 μM pyriliamine to eliminate the confounding effects of neural depolarizations and histamine release, respectively. Rings were then contracted with acetylcholine (an individual EC_50_ or EC_75_ calculated for each ring). Following the achievement of a stable contraction (typically 15 min), cumulatively increasing concentrations of dopamine (1 μM – 100 μM), the dopamine D_1_-like receptor agonist A68930 (1 μM - 100 μM) or the β-adrenoceptor agonist isoproterenol (0.5 nM – 1 μM) was incrementally added to the buffer in the baths. Control-contracted rings received vehicle (KH buffer) to serve as time controls for the A68930-induced relaxation. In separate experiments, rings were pretreated for 15 min with the dopamine D_2_ receptor selective antagonist L-741626 (10 μM), PKA inhibitor Rp-cAMPS (100 μM), large-conductance calcium-activated potassium (BK_Ca_) channel blocker iberiotoxin (100 nM) or the Epac antagonist NSC45576 (250 μM) before the addition of cumulatively increasing concentrations of A68930 (1 μM – 100 μM) or isoproterenol (0.5 nM – 1 μM) to assess their effects on airway smooth muscle force.

### Statistical analysis

Statistical analysis was performed using repeated measures of ANOVA, followed by Bonferroni posttest comparison using GraphPad Instat 3.0.6 software (GraphPad Software Inc., San Diego, CA). Data are presented as mean ± SEM; *P* < 0.05 was considered significant.

## Results

### RT-PCR analysis of dopamine D_1_-like receptors in human airway smooth muscle

**Figure 1:**
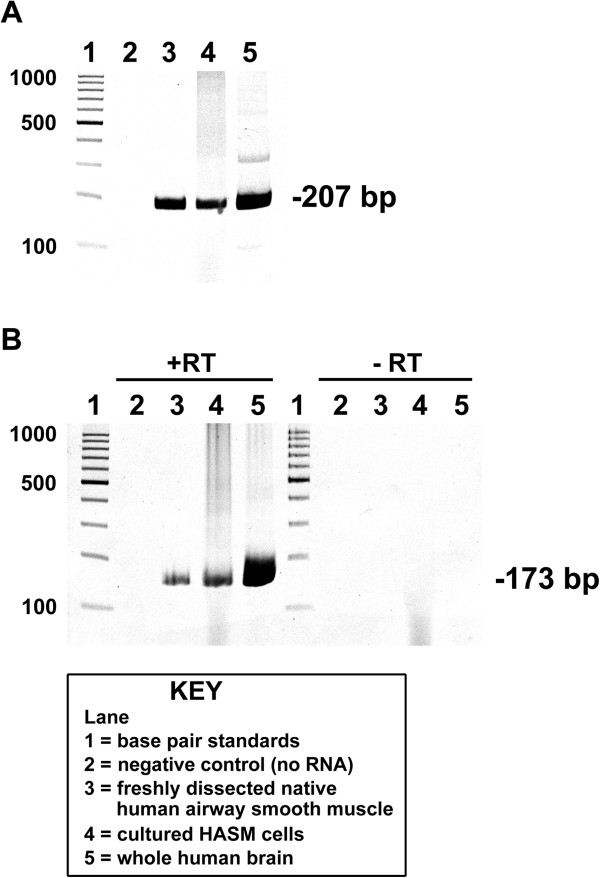
**RT-PCR analysis of dopamine D**_**1**_**-like receptors in human airway smooth muscle.** Representative gel images of RT-PCR analysis of total RNA using primers specific for human dopamine D_1_ receptor **(A)** and D_5_ receptor **(B)**. Total RNA extracted from freshly dissected human tracheal airway smooth muscle or primary cultures of human airway smooth muscle (HASM) cells was analyzed. Lanes 1: base pair standards; Lanes 2: negative control (no RNA); Lanes 3: total RNA from freshly dissected native human airway smooth muscle tissue; Lanes 4: total RNA from primary cultured HASM cells obtained from Lonza (Walkersville, MD); Lanes 5: total RNA from whole human brain. −RT; cDNA synthesis reactions performed in the absence of reverse transcriptase confirming that PCR products were not arising from contaminating genomic DNA.

RT-PCR analysis demonstrated the expression of mRNA encoding dopamine D_1_-like receptors (D_1_ and D_5_) in both freshly dissected native human airway smooth muscle tissue from the upper airways and in primary cultures of HASM cells from Lonza (Walkersville, MD) (Figure 1). The set of primers to amplify the cDNA of the “D_1_ subgroup (D_1_ and D_5_) of dopamine receptors are shown in Table 1. Total RNA from whole human brain (Clontech, Mountain View, CA) was used as a positive control. When cDNA was prepared in the absence of reverse transcriptase (-RT controls), no dopamine D_5_ receptor PCR products were obtained confirming that PCR products were arising from cDNA and not from contaminating genomic DNA (Figure 1B, right).

### Immunoblot analysis of dopamine D_1_-like receptors in human and guinea pig airway smooth muscle

**Figure 2:**
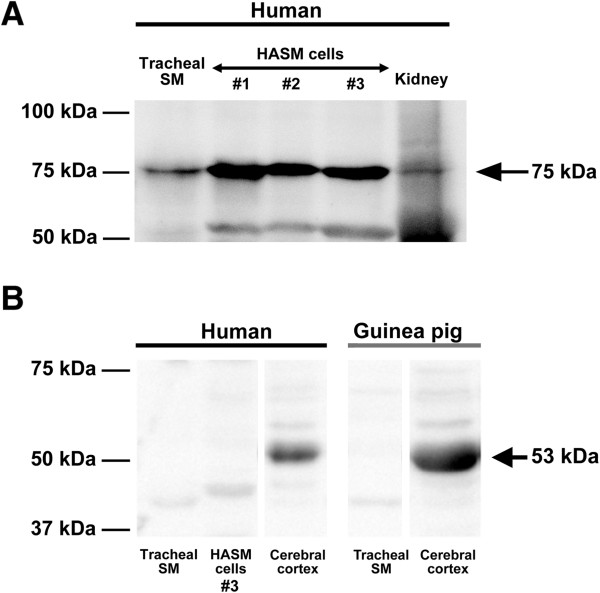
**Immunoblot analysis of dopamine D**_**1**_**-like receptors in human and guinea pig airway smooth muscle.** Representative immunoblot analyses using antibodies against the dopamine D_1_ receptor **(A)** and D_5_ receptor **(B)** using 100 μg total protein prepared from freshly dissected native human tracheal airway smooth muscle (SM), cultured HASM cells from all three different sources [#1: cultures of immortalized HASM cells gifted from Dr. William Gerthoffer, #2: primary cultured cells gifted from Dr. Reynold A. Panettieri, Jr., and #3: primary cultured cells obtained from Lonza (Walkersville, MD)], freshly dissected native guinea pig tracheal SM, human kidney, and human or guinea pig brain cerebral cortex. White spaces between the lanes in **(B)** indicate that these lanes were located on the same immunoblot but were not located in neighboring lanes on the original gel and immunoblot image.

A single immunoreactive band of expected molecular mass of approximately 75 kDa for dopamine D_1_ receptor was identified in freshly dissected native human airway smooth muscle, cultured HASM cells from all three different sources [#1: cultures of immortalized HASM cells gifted from Dr. William Gerthoffer, #2: primary cultured cells gifted from Dr. Reynold A Panettieri, Jr., and #3: primary cultured cells obtained from Lonza (Walkersville, MD)], and in human whole kidney (positive control) (Figure 2A). In contrast, no immunoreactive bands of appropriate molecular mass of approximately 53 kDa for dopamine D_5_ receptor was identified airway smooth muscle in freshly dissected native human and guinea pig airway smooth muscle tissue and cultured HASM cells except in whole brain extracted from human and guinea pig which were used as a positive control (Figure 2B).

### Immunohistochemical detection of dopamine D_1_ receptor expression in human and guinea pig airway smooth muscle

**Figure 3:**
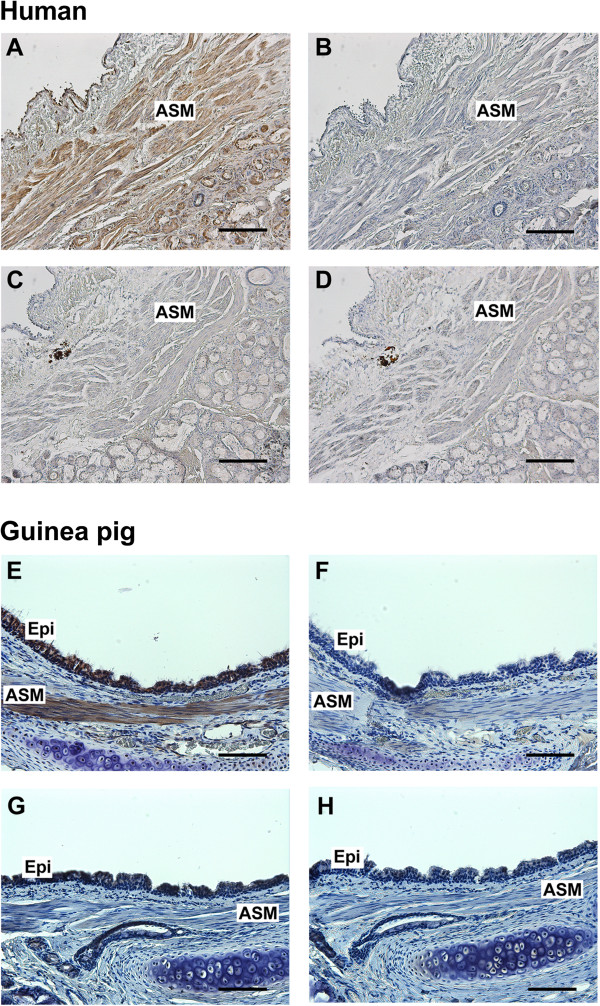
**Immunohistochemical detection of dopamine D**_**1 **_**receptor expression in human and guinea pig airway smooth muscle. (A** and **C)** Representative immunohistochemical staining of dopamine D_1_ receptor **(A)**, and dopamine D_5_ receptor **(C)** in paraformaldehyde/glutaraldehyde-fixed human trachea. **(B** and **D)** Anti-rabbit IgG isotype negative control in serial section of human tracheal airway smooth muscle. **(E** and **G)** Representative immunohistochemical staining of dopamine D_1_ receptor **(E)**, and dopamine D_5_ receptor **(G)** in paraformaldehyde-fixed guinea pig trachea. **(F** and **H)** Anti-rabbit IgG isotype negative control in serial section of guinea pig trachea. All sections were counterstained with hematoxylin. Calibration bar: 100 μm. ASM, airway smooth muscle; Epi, airway epithelium. Images are representative of at least 3 independent immunohistochemical analyses from human and guinea pig trachea.

To confirm the localization of dopamine D_1_ and D_5_ receptor protein in airway smooth muscle, immunohistochemistry was performed using a rabbit monoclonal antibody that recognizes the dopamine D_1_ receptor protein and a rabbit polyclonal antibody that recognizes the dopamine D_5_ receptor protein in paraffin sections of both human and guinea pig tracheal rings. Specific immunohistochemical staining of the dopamine D_1_ receptor was obtained throughout the smooth muscle layer of both human and guinea pig trachea (indicated by brown color) (Figure 3A and 3E). Consecutive sections processed with the isotype-specific rabbit IgG negative control primary antibody produced no staining (Figure 3B and 3F). In contrast, no staining of dopamine D_5_ receptor was obtained in the smooth muscle layer (Figure 3C and 3G), and consecutive sections processed with the isotype-specific rabbit IgG negative control primary antibody produced no staining (Figure 3D and 3H).

### Dopamine D_1_ receptor agonist-induced cAMP activity in human airway smooth muscle cells

**Figure 4:**
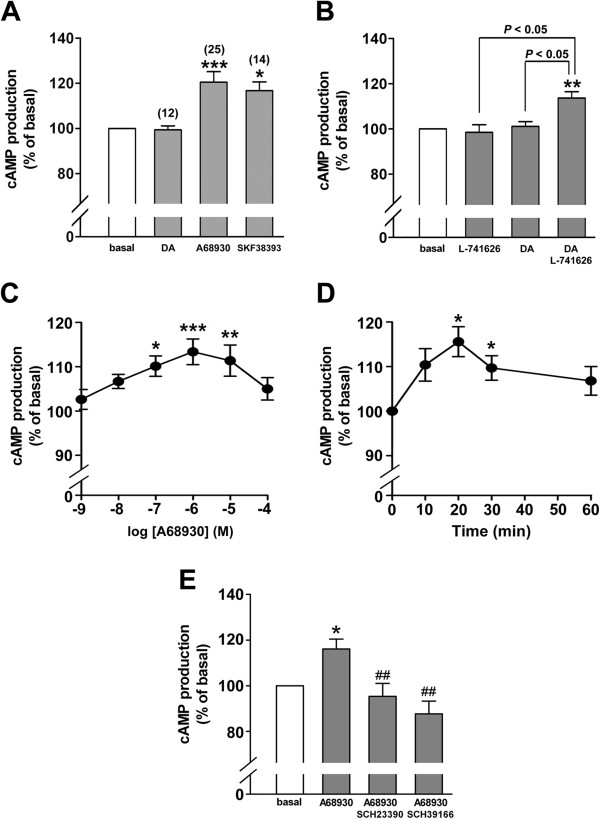
**Dopamine D**_**1 **_**receptor agonist-induced cAMP activity in human airway smooth muscle cells. (A)** The effects of dopamine (DA; 1 μM) or the dopamine D_1_-like receptor agonists (A68930 or SKF38393; 1 μM respectively) on cAMP production in cultured HASM cells. Number of experiments was shown in parentheses. **(B)** The effects of dopamine (1 μM) on cAMP production in the presence or absence of the dopamine D_2_ receptor antagonist (L-741626; 1 μM for 30 min pretreatment before dopamine treatment). N = 4. **(C)** Concentration-dependent effect of dopamine D_1_-like receptor agonist A68930 (1 nM - 100 μM) on cAMP production in cultured HASM cells. The cells were incubated with A68930 for 20 min before cAMP assay. N = 4 - 13. **(D)** Time-course effect of A68930 (1 μM) on cAMP production in cultured human airway smooth muscle cells. N = 6. **(E)** The effect of dopamine D_1_-like receptor selective antagonists SCH23390 or SCH39166 on A68930-stimulated cAMP production in HASM cells. Cells were pretreated with SCH23390 (1 μM) or SCH39166 (1 μM) for 30 min pretreatment prior to A68930 (1 μM) treatment for 20 min. Data represent means ± SEM. N = 7 - 9. *P < 0.05, **P < 0.01 and ***P < 0.001 compared with basal. ^##^*P <* 0.01 compared with A68930 alone. In each experiment, values were determined in triplicate.

Dopamine D_1_ receptor-mediated production of cAMP is well known in neurons. To examine whether dopamine D_1_ receptor agonists stimulate cAMP production in cultured HASM cells, we measured cAMP activity in the presence or absence of dopamine (1 μM) or the dopamine D_1_ receptor agonists (A68930 or SKF38393) (1 μM). Both dopamine D_1_-like receptor agonists significantly stimulated cAMP synthesis (A68930; *P* < 0.001, n = 25; SKF38393; *P* < 0.05, n = 14), while dopamine did not exert any effect (*n.s.*, n = 12) (Figure 4A). However, when the cells were pretreated with the dopamine D_2_ receptor antagonist L-741626 (1 μM) for 30 min, dopamine (1 μM) significantly stimulated cAMP synthesis (*P* < 0.01, n = 4), while L-741626 by itself did not exert any effect (Figure 4B). The dopamine D_1_-like receptor agonist A68930 significantly stimulated cAMP synthesis at concentrations ranging from 0.1 to 10 μM (*P* < 0.001 at 1 μM A68930, n = 13) (Figure 4C). A68930 (1 μM)-induced cAMP production peaked at 20 min and then slowly declined (Figure 4D). From these data, we chose the concentration of 1 μM A68930 and a duration of treatment of 20 min in the following experiments. A68930 (1 μM)-induced cAMP production was significantly blocked by pretreatment with the dopamine D_1_ receptor antagonists SCH23390 (1 μM) (*P <* 0.01, n = 9) or SCH39166 (1 μM) (*P <* 0.01, n = 7) (Figure 4E).

### Dopamine D_1_ receptor agonist induced airway smooth muscle relaxation after an acetylcholine EC_50_ contraction in intact guinea pig trachea

**Figure 5:**
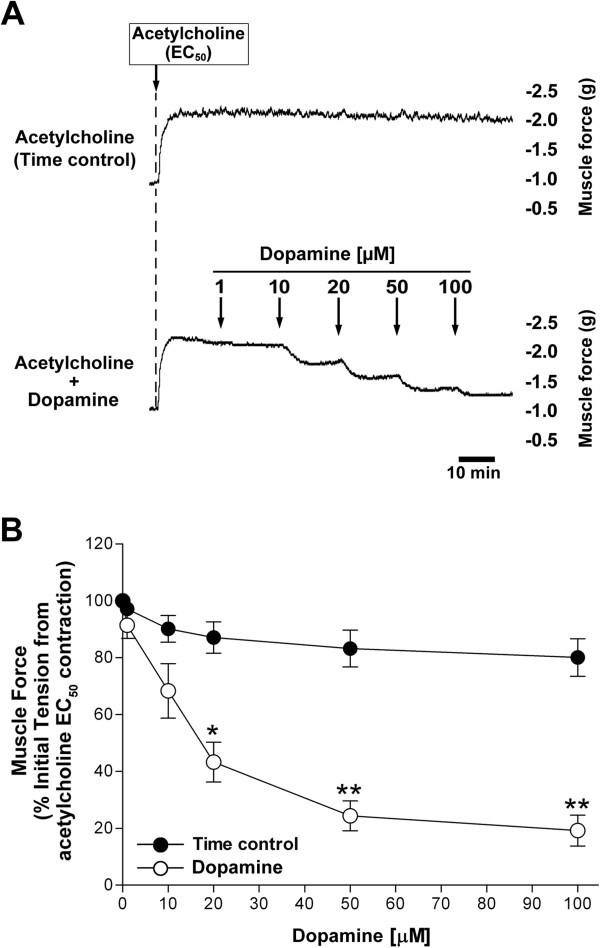
**Dopamine induced airway smooth muscle relaxation after an acetylcholine EC**_**50 **_**contraction in intact guinea pig trachea.** Dopamine, in a concentration-dependent manner, relaxes acetylcholine (Ach)-induced contractions in guinea pig tracheal rings. **(A)** Representative tension tracing in guinea pig tracheal ring illustrating relaxation of Ach (EC_50_) contraction by dopamine (1 - 100 μM). **(B)** The effect of cumulative concentration of dopamine (1 - 100 μM) on Ach (EC_50_)-induced guinea pig tracheal ring contraction. Open circles: dopamine alone. Filled circles: time control. Means ± SEM. *P < 0.05 and **P < 0.01 compared with time control. N = 5.

**Figure 6:**
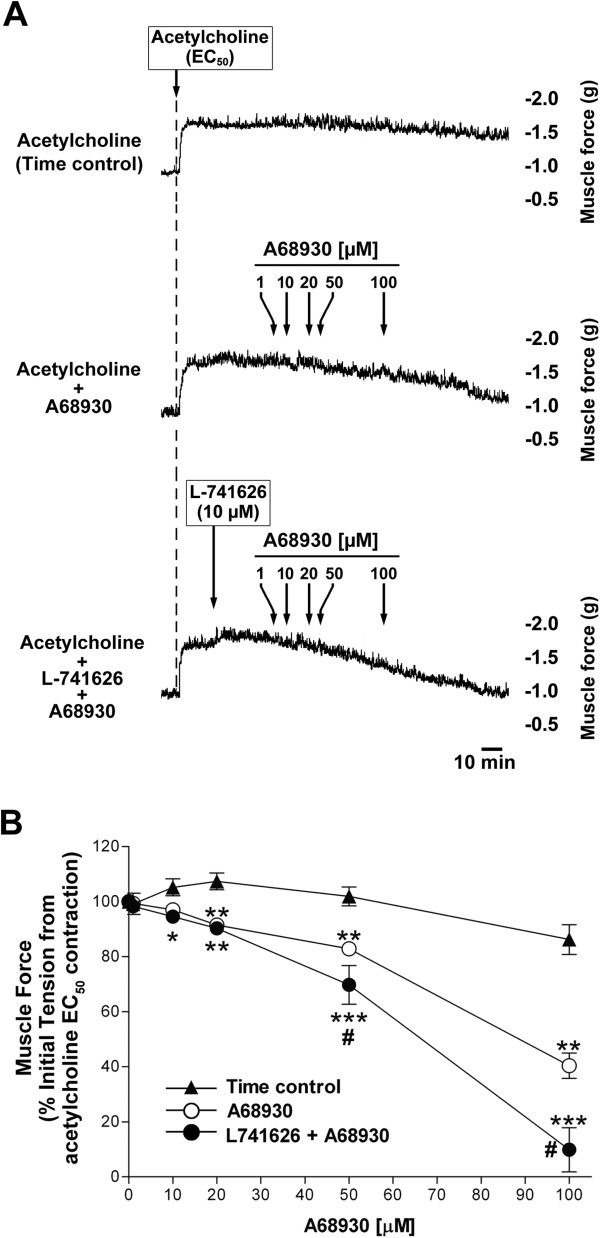
**Selective dopamine D**_**1 **_**receptor agonist induced airway smooth muscle relaxation after an acetylcholine EC**_**50 **_**contraction in intact guinea pig trachea.** The dopamine D_1_-like receptor agonist, A68930, in a concentration-dependent manner, relaxes Ach-induced contractions in guinea pig tracheal rings. **(A)** Representative tension tracing in guinea pig tracheal ring illustrating relaxation of Ach (EC_50_) contraction by A68930 (1 - 100 μM) in the presence or absence of dopamine D_2_ receptor selective antagonist L-741626 (10 μM). **(B)** The effect of cumulative concentration of A68930 (1 - 100 μM) on Ach (EC_50_)-induced guinea pig tracheal ring contraction in the presence or absence of L-741626 (10 μM). Open circles: A68930 alone. Filled circles: L-741626 + A68930. Filled triangle: time control. Means ± SEM. *P < 0.05, **P < 0.01 and ***P < 0.001 compared with time control. ^#^*P* < 0.05 compared with A68930 alone. N = 6.

Molecular identification of the dopamine D_1_ receptor, which stimulates cAMP production on airway smooth muscle, led us to question whether the receptor expressed on airway smooth muscle could modulate airway smooth muscle tone. Guinea pig tracheal rings contracted with an EC_50_ concentration of acetylcholine were significantly relaxed by dopamine (*P* < 0.05 compared with time control at 20 μM dopamine, and *P* < 0.01 compared with time control at 50 - 100 μM dopamine, n = 5) (Figure 5) and the dopamine D_1_-like receptor agonist A68930 (*P* < 0.01 compared with time control at 20 - 100 μM A68930, n = 6) (Figure 6). When guinea pig tracheal rings were contracted with higher concentrations of acetylcholine (EC_75_), A68930 (100 μM) still relaxed tracheal rings to the similar degree (n = 7) compared with its relaxation after contraction with an EC_50_ concentration of acetylcholine (n = 9) (34.83 ± 11.37% vs. 38.83 ± 12.35% of initial tension from acetylcholine EC_75_ and EC_50_ contraction, respectively, *n.s.*) (data not shown in figures). Although A68930 is a potent highly selective dopamine D_1_ receptor agonist (EC_50_ = 2.1 nM), it also acts as an weaker agonist on the dopamine D_2_ receptor at higher concentration (EC_50_ = 3.92 μM) [22]. To confirm A68930-induced airway relaxation is not mediated via dopamine D_2_ receptor, we used the dopamine D_2_ receptor selective antagonist L-741626. Interestingly, under identical conditions, the pretreatment of rings with L-741626 (10 μM) significantly augmented the airway relaxation induced by A68930 (*P* < 0.05 compared with A68930 alone at 50 - 100 μM A68930, n = 6) (Figure 6).

### Protein kinase A (PKA) is involved in Dopamine D_1_ receptor agonist-induced guinea pig airway smooth muscle relaxation

**Figure 7:**
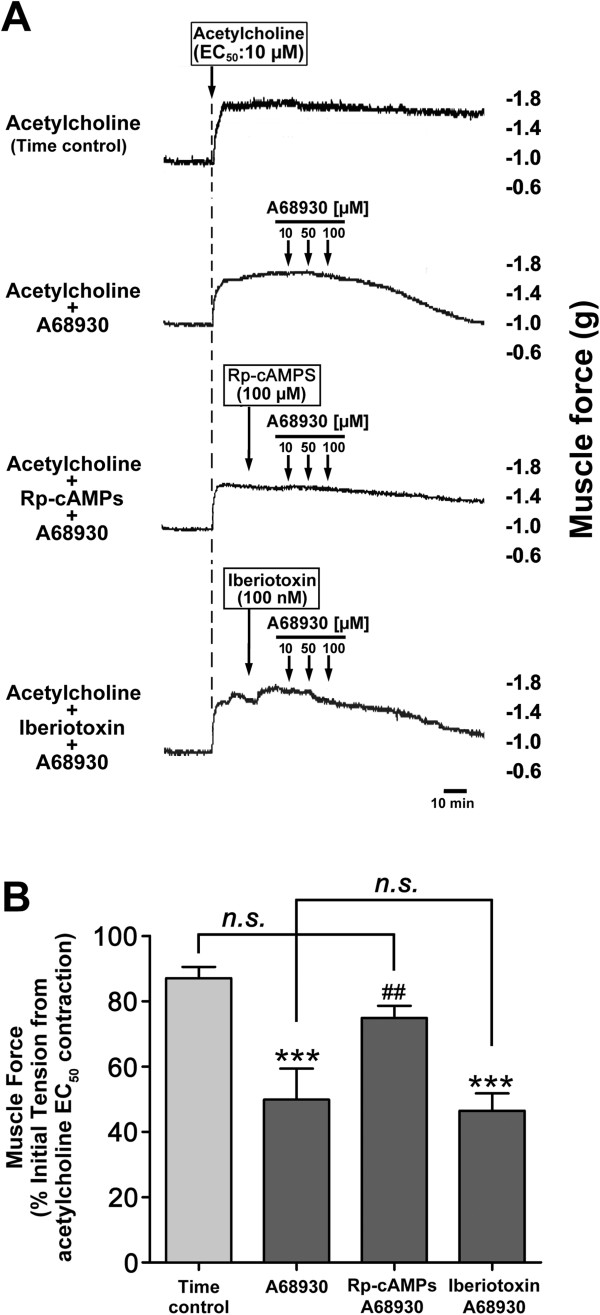
**PKA is involved in dopamine D**_**1 **_**receptor agonist-induced airway smooth muscle relaxation. (A)** Representative tension tracing in guinea pig tracheal ring illustrating the effect of pretreatment with the PKA inhibitor Rp-cAMPS (100 μM) or BK_Ca_ channel blocker iberiotoxin (100 nM) on relaxation of Ach (EC_50_) contraction by the dopamine D_1_-like receptor agonist A68930 (10 - 100 μM). **(B)** The effect of pretreatment with the PKA inhibitor Rp-cAMPS (100 μM) or BK_Ca_ channel blocker iberiotoxin (100 nM) on A68930 (100 μM)-mediated tracheal ring relaxation. Means ± SEM. ***P < 0.001 compared with time control. ^##^*P* < 0.01 compared with A68930 alone. N = 6.

Classically, activation of PKA by cAMP facilitates airway smooth muscle relaxation [23]. To examine the role of PKA in regulating dopamine D_1_ receptor-mediated airway smooth muscle relaxation, we used Rp-cAMPS as a PKA inhibitor. The pretreatment of guinea pig airway rings with Rp-cAMPS (100 μM) significantly attenuated the relaxation induced by A68930 (*P* < 0.01 compared with A68930 alone, n = 6) (Figure 7). In contrast, blockade of the BK_Ca_ channel with iberiotoxin did not inhibit the relaxation induced by A68930 (*n.s.* compared with A68930 alone, n = 6) (Figure 7).

### Dopamine D_1_ receptor-mediated guinea pig airway relaxation is Epac independent

**Figure 8:**
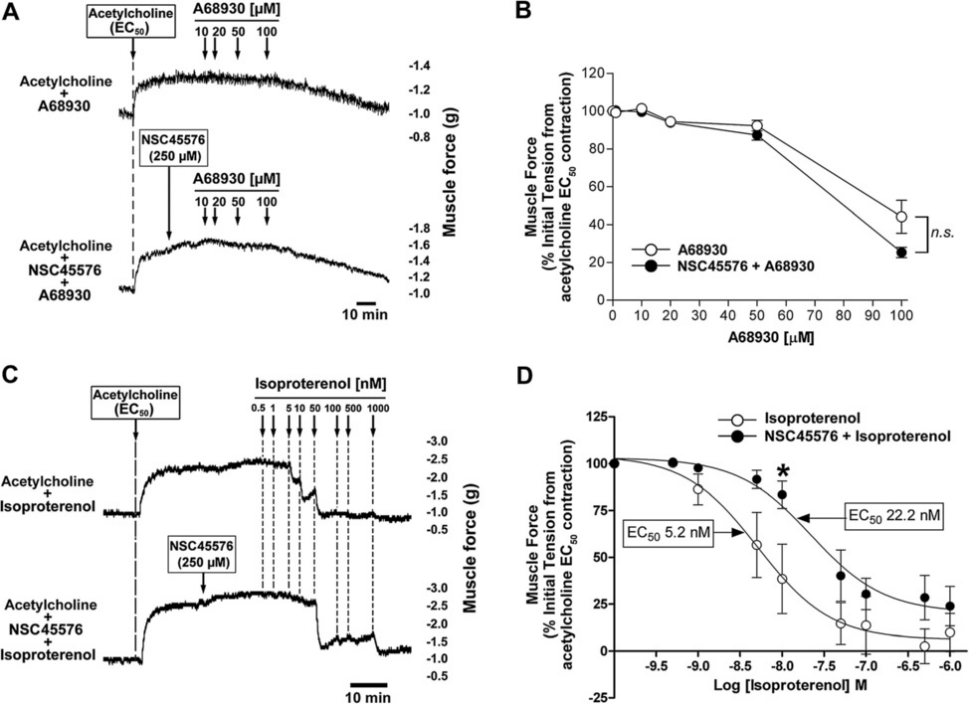
**Dopamine D**_**1 **_**receptor-mediated guinea pig airway relaxation is Epac independent. (A)** Representative tension tracing in guinea pig tracheal ring after an acetylcholine contraction followed by increasing concentrations of the dopamine D_1_-like receptor agonist A68930 (1 - 100 μM) in the presence or absence of the Epac antagonist NSC45576 (250 μM). Top tracing is A68930 alone, and bottom tracing represent treatment with A68930 after 15 min pretreatment with NSC45576. **(B)** The effect of pretreatment with NSC45576 (250 μM) on A68930 (1 - 100 μM)-mediated tracheal ring relaxation. Open circles: A68930 alone. Filled circles: NSC45576 + A68930. Means ± SEM. N = 4. **(C)** Representative tension tracing in guinea pig tracheal ring after an acetylcholine contraction followed by increasing concentrations of β-adrenoceptor agonist isoproterenol in the presence or absence of NSC45576 (250 μM). Top tracing is isoproterenol alone, and bottom tracing represent treatment with isoproterenol after 15 min pretreatment with NSC45576. **(D)** Isoproterenol concentration-response curves comparing treatment with isoproterenol alone (open circles) to isoproterenol after pretreatment with NSC45576 (250 μM) (filled circles). *P < 0.05 compared with isoproterenol alone. N = 5.

Recently, Epac has been recognized as an alternative cAMP effector able to elicit airway smooth muscle relaxation [17]. To examine whether Epac is involved in dopamine D_1_ receptor-meidated airway relaxation, Epac antagonist NSC45576 was used. The pretreatment of guinea pig airway rings with NSC45576 (250 μM) did not block A68930-induced airway relaxation (*n.s.* compared with A68930 alone, n = 4) (Figure 8A and 8B). Isoproterenol, which is considered to exert airway smooth muscle relaxation in part through Epac [17], was used to confirm that NSC45576 was effective as an Epac antagonist in guinea pig airway smooth muscle. NSC45576 resulted in a significant rightward shift in the isoproterenol relaxation concentration-response curve compared with treatment with isoproterenol alone [EC_50_ = 22.2 nM vs. 5.2 nM, respectively; *P* < 0.05 at 10 nM isoproterenol, n = 5] (Figure 8C and 8D).

## Discussion

The major finding of the present study is that functional dopamine D_1_ receptors are expressed in both human and guinea pig airway smooth muscle. The dopamine D_1_ receptors were detected at the level of mRNA and protein, and a dopamine D_1_-like receptor-selective agonist facilitated relaxation against acetylcholine-induced contraction in airway smooth muscle through cAMP production and PKA activation.

In addition to the expression in the central nervous system, numerous studies have also demonstrated the expression of functional dopamine D_1_ receptors in peripheral organs and tissues such as the myocardium, pulmonary artery, renal tubules, gastrointestinal tract, and the zona glomerulosa cells of the adrenal cortex [4-7,24-27]. Identification of mRNA in freshly dissected human airway smooth muscle ensures that native expression of the dopamine D_1_-like receptor (D_1_ and D_5_) is being examined. However, a limitation of this approach is that even with careful dissection, freshly isolated tissue could be contaminated by other cells types that could give rise to cDNA products during sensitive RT-PCR amplification. To confirm the expression of dopamine D_1_–like receptor in airway smooth muscle itself, we also evaluated its expression in primary cultured HASM cells which are a homogenous population of airway smooth muscle cells. In the present study, there was good agreement between the expression of dopamine D_1_-like receptor in freshly isolated native airway smooth muscle and from cultured HASM cells. These findings were in accordance with the previous findings using expression microarrays in that mRNA of both dopamine D_1_-like receptor subtypes was expressed in HASM cells [28]. Moreover, in agreement with these mRNA analyses, the dopamine D_1_ receptor protein was also identified in airway smooth muscle from native human airway smooth muscle, and cultured HASM cells from three independent sources. Immunohistochemical analysis also confirmed the expression of dopamine D_1_ receptor on airway smooth muscle in both human and guinea pig airway smooth muscle. On the other hand, the dopamine D_5_ receptor protein was not identified in both human and guinea pig native airway smooth muscle tissue, and cultured HASM cells by immunoblotting and immunohistochemistry. The discrepancy of the results obtained from RT-PCR and protein analyses on the expression of dopamine D_5_ receptor on airway smooth muscle is likely due to posttranscriptional regulation, differences in mRNA and protein turnover rates [29,30], or detection of small amounts of dopamine D_5_ mRNA by sensitive PCR techniques that does not translate into detectable levels of dopamine D_5_ protein. Several studies also described the correlation between mRNA and protein expression as moderately or weakly positive with correlation coefficients ranging from 0.2 to 0.5 [30-33].

Molecular identification of dopamine D_1_ receptor on airway smooth muscle itself led us to question whether functional dopamine D_1_ receptors could modulate airway smooth muscle tone. First, we sought to assess its ability to increase cyclic AMP and relax airway smooth muscle. Many studies have demonstrated that the activation of G_s_-coupled receptors (e.g., β_2_-adrenoceptor) stimulates adenylyl cyclase activity, which leads to cAMP accumulation in airway smooth muscle [15,34,35]. Accumulation of cAMP content in airway smooth muscle facilitates relaxation [15,34,35]. In accordance with the previous findings on G_s_-coupled receptors, our findings demonstrate that activation of the dopamine D_1_ receptor expressed on HASM cells with the endogenous ligand dopamine stimulated cAMP production in HASM cells but only when the dopamine D_2_-G_i_ coupled receptor was inhibited with L-741626. These findings suggest that dopamine exerts its effect equally on both dopamine D_1_-G_s_ coupled receptor and dopamine D_2_-G_i_ coupled receptor in HASM cells, and selective stimulation of the dopamine D_1_ receptor is required to stimulate cAMP production. Dopamine also relaxed acetylcholine-contracted guinea pig airway rings, however, contrary to the dopamine-stimulated cAMP production in HASM cells, the relaxation was produced without the blockade of dopamine D_2_ receptor (data not shown). These findings would suggest that the dopamine D_2_ receptor is more effectively coupled to G_i_/adenylyl cyclase inhibition in the cultured HASM cells compared to intact tracheal rings. Another possibility is that in tracheal rings the dopamine-mediated relaxation is partially mediated through dopamine effects on β_2_-adrenoceptors. It was previously reported that dopamine-mediated relaxation of acetylcholine-contracted guinea pig trachea was partially blocked by the β-adrenoceptor antagonist propranolol [12]. These findings suggest that dopamine induces airway smooth muscle relaxation via both dopamine D_1_ receptors and β_2_-adrenoceptors.

To characterize the dopamine D_1_ receptor-specific effects on airway smooth muscle, the selective dopamine D_1_ receptor agonists A68930 and SKF38393 were used in the present study. Both agonists stimulated cAMP production in HASM cells, and A68930 induced airway smooth muscle relaxation in guinea pig tracheal rings. The specificities of A68930 (1 μM) at the dopamine D_1_ receptor in the present study were further supported by the finding that two different dopamine D_1_-like receptor antagonists SCH23390 or SCH39166 significantly blocked the effects of A68930 on cAMP production. However, treatment the HASM cells with A68930 at a higher concentration (100 μM) resulted in decrease in cAMP production compared with 1 μM of A68930. A68930 is a potent dopamine D_1_ receptor agonist (EC_50_ = 2.1 nM), but it also stimulates the G_i_-coupled dopamine D_2_ receptor at higher concentrations (EC_50_ = 3.92 μM) [22]. We have previously reported that the acute activation of the dopamine D_2_ receptor expressed in HASM cells inhibited forskolin-stimulated adenylyl cyclase activity [14], which consequently reduces in cellular cAMP levels. These findings indicate that A68930 at higher concentrations may lose its selectivity against dopamine D_1_ receptors. This idea was further supported in the present study by demonstrating that the dopamine D_2_ receptor selective antagonist L-741626 significantly augmented A68930-induced guinea pig airway ring relaxation. In the present study, the concentration of A68930 which was required to produce significant relaxation in guinea pig airway ring was higher (≧ 20 μM) than that required to produce significant cAMP production in HASM cells (≧ 100 nM). Previous reports in tissue organ bath also showed that phenylephrine-contracted human corpus cavernosum smooth muscle strip without endothelium suspended in organ bath was dose-dependently relaxed by A68930 (EC_50_ = 3.5 ± 0.9 μM) [36]. In contrast, in a cell-based cAMP assay, the EC_50_ value of A68930-stimulated cAMP activity in LLC-PK_1_ cells was much lower (EC_50_ = 12.7 ± 2.7 nM) [37]. These results and our findings suggest that at least 200-fold higher concentration of A68930 is needed for experiments on tissues where penetration to their site of action may not be equivalent to that achieved in a cell monolayer.

Classically, most cAMP effects have been attributed to the activation of PKA. PKA has multiple intracellular targets that favor relaxation including phosphorylation of MLCK which leads to a decrease in its affinity for the calcium-calmodulin complex [38], resulting in airway smooth muscle relaxation [15,16]. As expected, the present study demonstrated that a PKA inhibitor Rp-cAMPS significantly attenuated dopamine D_1_ receptor-mediated airway relaxation. However, several cAMP-mediated cellular events in airway smooth muscle are insensitive to PKA inhibition. Spicuzza *et al.* suggested that β_2_-adrenoceptor agonists suppressed acetylcholine-induced guinea pig airway contraction by mechanisms that are independent of PKA [39]. Recently, Epac has been identified as cAMP-regulated guanine nucleotide exchange factors for Ras-like GTPases, such as Rap1 and Rap2 [40], and been recognized as an alternative cAMP sensor to relax smooth muscle in addition to PKA. Roscioni *et al.* demonstrated the protein expression of two isoforms of Epac (Epac 1 and Epac2) in human and guinea pig trachea, and activation of Epac relaxes airway smooth muscle by decreasing MLC phosphorylation [17]. They also claimed that β_2_-adrenoceptor agonist relaxes airway smooth muscle through Epac rather than PKA [17]. In contrast, in the present study, the Epac selective antagonist NSC45576 did not block dopamine D_1_ receptor agonist-induced airway relaxation. These results suggest that although both the dopamine D_1_ receptor and β_2_-adrenoceptor are G_s_-coupled receptor, induce airway smooth muscle relaxation and increase cAMP, that the subsequent signaling targets of this cAMP elevation may not be identical following these receptors’ activations.

In addition to these cAMP-dependent mechanisms, a mechanism independent of cAMP is also relevant in β_2_-adrenoceptor-mediated relaxation of airway smooth muscle [23,41], which involves the opening of the BK_Ca_ channel causing plasma membrane hyperpolarization facilitating relaxation [41,42]. However, in the present study, blockade of the BK_Ca_ channel with iberiotoxin did not inhibit relaxation induced by a dopamine D_1_ receptor agonist.

In the present study, there are several differences in the results from the cell-based cAMP assays and intact tissue organ bath studies with the dopamine D_1_ receptor agonist A68930. The first difference is that the relaxation evoked by isoproterenol and dopamine was rapid, while that evoked by A68930 was slow. These results suggest that the agonists’ interaction with the receptor can affect the speed of onset and duration of the induced relaxation. Variations in the molecular structure of agonists can affect the onset and duration of bronchodilation. For example, isoproterenol is a short-acting β agonist and has rapid onset, while the long-acting β_2_-adrenoceptor agonist salmeterol causes sustained relaxation in airway smooth muscle of at least 12 hrs after a single administration [43], but the speed of onset is much slower than short-acting agonists such as isoproterenol [44]. There are remarkable differences in molecular structure between these agonists and these differences affect the speed of onset and duration of their effects [45,46]. These findings suggest that, even though the agonists act on the same receptor, the molecular structure can affect the kinetics of the drug effect at the receptor. The second difference between cell-based and tissue-based results with A68930 in the present study is the time required to reach significant relaxation in guinea pig airway rings was longer than the time required for significant stimulation of cAMP production in HASM cells. The dopamine D_1_ agonists A68930 and A77636 contain an isochroman structure and are potent dopamine D_1_ agonists which exert long-lasting effects compared with dopamine *in vivo*. For example, A68930 elicited prolonged contralateral turning behavior in 6-hydroxydopamine lesioned rats (>20 hrs) [47]. In contrast, several reports showed that A68930 stimulated cAMP production within 15 - 20 min [37,48]. However, it has not been determined whether the long-acting effects of A68930 rely exclusively on cAMP production. Thus, it is possible that sustained relaxation of airway smooth muscle is not solely mediated by increased cAMP and that other intracellular signaling events may be involved. Further studies will be needed to clarify the intracellular mechanisms by which A68930 induces airway smooth muscle relaxation.

## Conclusion

In summary, our findings demonstrated for the first time the molecular expression of G_s_-coupled dopamine D_1_ receptors in human and guinea pig airway smooth muscle that can stimulate cAMP production in HASM cells and relax intact airway smooth muscle contracted with acetylcholine in the PKA-dependent manner. Although the G_s_-coupled β_2_-adrenoceptor is a critical mediator of therapeutic relaxation of airway smooth muscle in diseases such as asthma and chronic obstructive pulmonary disease, less attention has focused on other G_s_-coupled receptors on airway smooth muscle. Our findings suggest that the G_s_-coupled dopamine D_1_ receptor may be a potential therapeutic target for relaxation of airway smooth muscle.

## Competing interests

The authors declare that they have no competing interests.

## Authors’ contributions

KM and CWE conceived and designed these studies; KM, YZ, DX, FM, FD and CWE performed experiments; KM, YZ, DX, FM, and CWE analyzed data; KM, YZ, DX, FM, and CWE interpreted results of experiments; KM and CWE prepared figures; KM, EM, and CWE drafted manuscript; KM, EM, and CWE edited and revised manuscript; All authors read and approved the final version of manuscript.
